# Chemical Composition and Antioxidant Activity in Different Tissues of *Brassica* Vegetables

**DOI:** 10.3390/molecules20011228

**Published:** 2015-01-13

**Authors:** Shiva Ram Bhandari, Jung-Ho Kwak

**Affiliations:** Vegetable Research Division, National Institute of Horticultural and Herbal Science, Rural Development Administration, Suwon 440-706, Korea; E-Mail: shivarbhandari@gmail.com

**Keywords:** antioxidant activity, *Brassica*, fatty acids, free sugar, glucosinolate, total flavonoid

## Abstract

This research was conducted to evaluate glucosinolate profiles, vitamin C, total phenol, total flavonoid, and free sugar (glucose, fructose, and sucrose) content, fatty acid composition, and antioxidant activity in floret and leaf of six cauliflower and broccoli cultivars. The level of chemical constituents as well as antioxidants significantly varied among crop types, cultivars, and their different parts, in that phytochemicals such as glucosinolate were statistically higher in florets compared with leaves in both broccoli and cauliflower cultivars. In contrast, total flavonoid and free sugar were found at higher levels in the leaf parts. The Asia purple cultivar exhibited statistically higher vitamin C (649.7 mg·100 g^−1^), total phenol (1345.2 mg·GAE 100 g^−1^), and total flavonoid (632.7 mg·CE 100 g^−1^) contents and consequently had the highest antioxidant activity (1.12 mg·mL^−1^) in its florets, while Baeridom and Bridal had the highest total glucosinolate (9.66 µmol·g^−1^) and free sugar (318.6 mg·g^−1^) contents, respectively compared with other cultivars. Likewise, the major fatty acids were palmitic (23.52%–38.42%), linoleic (13.09%–18.97%), and linolenic (26.32%–51.80%) acids, which comprised the highest compositional ratio (more than 50%) of polyunsaturated fatty acids (PUFAs) in most cultivars. Among the antioxidants, total phenol exhibited the most significant positive correlation (*r* = 0.698 **) with antioxidant activity, followed by vitamin C (*r* = 0.522 **) and total flavonoid (*r* = 0.494 **), indicating their significant contributions to total antioxidant activity.

## 1. Introduction

*Brassica* vegetables such as cauliflower and broccoli are popular and are among the most consumed vegetables in the world. Many epidemiological studies have indicated that a diet rich in these vegetables is associated with reduced risk of a several type of cancers, type 2 diabetes, and cardiovascular diseases [1,2,3]. Additionally, *Brassicas* are known to possess antioxidant activity [4,5]. Such beneficial health properties of these crops are due to the presence of health-promoting compounds such as vitamins, carotenoids, phenols, flavonoids, minerals, and glucosinolates [6,7,8,9,10]. Among these, glucosinolates are one of the most important phytochemicals in *Brassica* crops, a large group of sulfur-containing compounds possessing anticancer activity that are known to be responsible for the pungent flavor of the plants [11,12]. Vitamin C is another health-promoting water-soluble primary nutrient in broccoli and other *Brassica* crops [13] that protects against cell death, singlet oxygen, and hydroxyl radicals and acts as a lipid peroxidation chain-breaking agent [14]. Likewise, polyphenols are a large group of antioxidant compounds present in considerable amounts in these vegetables [15], often considered the most abundant antioxidants in the human diet [2]. Flavonoids and their derivatives are the largest and most prominent group of polyphenols and are ideal scavengers of peroxyl radicals due to their specific reduction actions relative to alkyl peroxyl radicals, making them effective inhibitors of lipoperoxidation [16]. Likewise, free sugars in *Brassica* crops are responsible for masking the bitter taste of glucosinolates, promoting human consumption [17]. Additionally, the lipophilic portion of these plant extracts may also contain both saturated fatty acids (SFA) and unsaturated fatty acids (monounsaturated fatty acids (MUFA) and polyunsaturated fatty acids (PUFA)), which contribute to human health in many ways [18,19]. 

The content of these compounds in *Brassica* vegetables varies significantly depending on the genotypes of cultivars, the specific plant tissue, fertilization, growing season, and several other environmental factors [9,20,21,22,23]. For example, significant variation in glucosinolate concentration as well as the content of other compounds has been shown in the same cultivar grown in different seasons [6,24]. There are several reports regarding the glucosinolate, vitamin C, phenolic, and flavonoid content in various *Brassica* crops such as cauliflower and broccoli [6,9,15,25]. However, most of this research has focused only on their floret parts, and information about such compounds in leaf tissue is scarce. Furthermore, information regarding fatty acid profiles is very limited, and most previous research has been related only to the seeds [26]. It is therefore important to establish the compositional ratio of fatty acids in different cauliflower and broccoli cultivars, as fatty acids may play an important role in human health. Therefore, in this study, we aimed to evaluate the influence of plant parts on the health benefits of some chemical constituents such as glucosinolates, vitamin C, phenolics, flavonoids, and free sugar content, along with the fatty acid composition and antioxidant activity of commercial cauliflower and broccoli cultivars. 

## 2. Results and Discussion

### 2.1. Vitamin C, Total Phenol, and Total Flavonoid Content 

**Figure 1:**
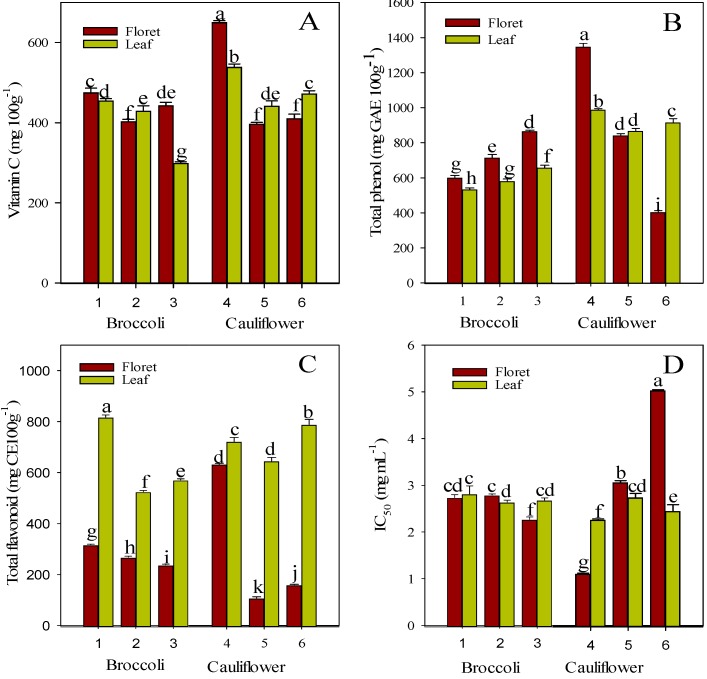
Vitamin C (**A**); total phenol (**B**); total flavonoid (**C**); and antioxidant activity (**D**) in floret and leaf of broccoli and cauliflower cultivars. Each vertical bar represents mean ± SD of three independent replications; different letters within a box indicate a statistically significant difference at *p* < 0.05 by Duncan’s multiple-range test. Broccoli cultivars: 1, AMaGi; 2, Baeridom; 3, Cheonjae. Cauliflower cultivars: 4, Asia purple; 5, Asia white; 6, Bridal.

Our results showed variation in antioxidant (vitamin C, total phenol, and total flavonoid) contents with crop type, cultivar, and plant part (Figure 1A–D). In broccoli, the vitamin C content in florets and leaves ranged from 402.8 to 474.7 mg·100 g^−1^ and from 298.6 to 454.3 mg·100 g^−1^, respectively. The cultivar AMaGi exhibited higher vitamin C content in floret and leaf tissues (Figure 1A) than did Baeridom and Cheonjae. The value observed in this study was lower than that found in a previous study by Koh* et al.* [27], who observed 573.5–1313.5 mg·100 g^−1^ of vitamin C in various commercial broccolis. In contrast, total phenol content was lower in AMaGi than in Baeridom and Cheonjae in both the floret (599.6 mg·GAE 100 g^−1^) and leaf (533.6 mg·GAE 100 g^−1^) parts (Figure 1B). Regardless of cultivars and plant tissue, total phenol content ranged from 533.6 mg·GAE 100 g^−1^ in AMaGi leaf tissue to 740.1 mg·GAE 100 g^−1^ in Cheonjae florets, which was higher than previously reported by Zhang and Hamauzu [28] and within the range reported by Koh* et al.* [27]: 481–1577 mg·GAE 100 g^−1^ of total phenol in various broccoli cultivars. All three cultivars showed statistically higher total phenol contents in florets compared with leaf tissue. In contrast, total flavonoid content was statistically higher in leaf tissue in all the cultivars and showed higher variation (more than twofold) between florets and leaves in all the cultivars. Similar to vitamin C, the highest flavonoid content was present in AMaGi in both floret (317.1 mg·CE 100 g^−1^) and leaf (816.8 mg·CE 100 g^−1^). The presence of higher total flavonoid content in leaf suggests higher nutritional value of leaves, as flavonoids possess strong antioxidant activity and inhibit oxidative stress [29].

The vitamin C content in floret tissues of the three cauliflower cultivars was highest in Asia purple (649.7 mg·100 g^−1^), followed by Bridal (410.4 mg·100 g^−1^) and Asia white (396.7 mg·100 g^−1^) (Figure 1A). This result was in agreement with Picchi* et al.* [30], who reported 346–638 mg·100 g^−1^ of vitamin C, though it was higher than the vitamin C content reported by Singh* et al.* [9]. Compared with the florets, the leaf parts showed statistically higher vitamin C content in Asia white (441.7 mg·100 g^−1^) and Bridal (471.9 mg·100 g^−1^). Likewise, total phenol content was highest in Asia purple (1345.2 mg·GAE 100 g^−1^) and lowest in Bridal in the floret part (Figure 1B). In contrast to the vitamin C content, only Bridal (914.1 mg·GAE 100 g^−1^) exhibited statistically higher total phenol content in leaves compared with florets. Similar to the broccoli cultivars, total flavonoid content in cauliflower cultivars was also statistically higher in leaf parts compared with floret parts, and Asia white and Bridal exhibited an approximately fivefold higher flavonoid content in leaf tissue compared with their respective floret parts. The presence of higher total flavonoid content in leaf parts in both broccoli and cauliflower crops suggest that its leaf can be used as an alternative source of total flavonoids, as flavonoids are responsible for various beneficial health effects [27,29,31].

### 2.2. Glucosinolate Profiles 

Total and individual glucosinolate concentrations were significantly affected by crop type, cultivar, and plant part in most cases (Table 1). Similar to the previous reports [25,32], glucoraphanin was the major glucosinolate in both parts of broccoli cultivars except in the leaf tissue of AMaGi, where glucobrassicin was the dominant glucosinolate (2.30 µmol·g^−1^) (Table 1). Furthermore, glucobrassicin was the second major glucosinolate except in the florets of AMaGi, ranging from 2.03 in AMaGI to 2.44 µmol·g^−1^ in Cheonjae florets and from 0.92 µmol·g^−1^ in Cheonjae to 2.30 µmol·g^−1^ in AMaGi leaves. All of the broccoli cultivars showed statistically higher total glucosinolate concentrations in the floret tissues compared with leaf tissues, and Baeridom (9.66 µmol·g^−1^) showed the highest total glucosinolate concentration compared with AMaGi (8.09 µmol·g^−1^) and Cheonjae (5.59 µmol·g^−1^) (Table 1). The glucosinolate levels found in this study were within the range found by Lee* et al.* [25] and lower than the results reported by Rosa and Rodrigues [32]. The composition of glucosinolate concentration in this study was also different than in Aries* et al.* [6], who reported considerable amount of glucoiberin in broccoli. Such differences in glucosinolate concentration and composition in this study might be due to the difference in genotype of the cultivars and other several environmental factors.

Among cauliflower cultivars, Asia purple exhibited the highest total glucosinolate concentration (8.80 µmol·g^−1^), followed by Bridal (7.41 µmol·g^−1^) and Asia white (1.97 µmol·g^−1^) (Table 1). With the exception of Asia white, the cultivars exhibited relatively higher total glucosinolates in their floret parts compared with leaf parts. Among the nine glucosinolates analysed in our experiment, five glucosinolates were detected in Asia purple, and Asia white and Bridal showed the presence of gluconapin and other glucosinolates. Similar to the results of Hodges *et al.* [33], glucobrassicin was a major glucosinolate compound in cauliflower, except in florets of Asia white (0.05 µmol·g^−1^), which revealed relatively higher proportions of glucoraphanin and gluconapin. However, the levels of gluconapin and glucoraphanin observed in this study appear to be higher than those in previous reports [30,33].

**Table 1:** Glucosinolate profile (µmol·g^−1^ in dry weight) in various parts of broccoli and cauliflower cultivars.

Crop	Cultivar	Part	Progoitrin	Glucoraphanin	Sinigrin	Gluconapin	Glucobrassicanapin	Glucobrassicin	Total Glucosinolates
Broccoli	AMaGi	Floret	2.73 ^x^ a ^y^	2.77 b	N/D ^z^	0.56 a	N/D	2.03 c	8.09 c
		Leaf	0.34 e	2.05 d	N/D	0.20 c	N/D	2.30 c	4.89 fg
	Baeridom	Floret	1.66 b	5.19 a	0.31 e	0.43 b	N/D	2.07 c	9.66 a
		Leaf	0.22 f	2.11 d	0.06 f	0.08 de	N/D	1.93 c	4.40 g
	Cheonjae	Floret	0.44 d	2.50 c	0.09 f	0.12 d	N/D	2.44 c	5.59 ef
		Leaf	0.04 h	1.10 e	0.02 f	0.04 ef	N/D	0.92 d	2.12 hi
Cauliflower	Asia purple	Floret	0.41 d	0.69 fg	1.71 a	N/D	0.85 a	5.14 a	8.80 b
		Leaf	0.13 g	0.32 h	1.04 c	N/D	0.43 b	5.28 a	7.20 d
	Asia white	Floret	0.51 c	0.65 fg	0.12 f	0.59 a	0.05 c	0.05 e	1.97 h
		Leaf	0.07 h	0.73 fg	0.44 d	N/D	0.42 b	1.15 d	2.81 h
	Bridal	Floret	0.31 e	0.76 f	1.17 b	0.09 de	N/D	5.08 a	7.41 cd
		Leaf	0.16 g	0.49 gh	0.98 c	0.11 d	0.41 b	3.69 b	5.84 e

^x^ Values are the mean of three independent replications; ^y^ Different letters within the column are statistically significant by Duncan’s multiple-range test at *p* < 0.05; ^z^ N/D: Not detected.

### 2.3. Free Sugar Content

Three free sugars, namely glucose, fructose, and sucrose, were evaluated in this study, and glucose was most abundant in all cultivars and their parts, followed by fructose and then sucrose (Table 2). Of all cultivars and plant parts, total free sugar content in broccoli ranged from 153.3 mg·g^−1^ in the florets to 241.6 mg·g^−1^ in the leaves of AMaGi, and total sugar content was statistically higher in leaf parts, except in Cheonjae (237.2 mg·g^−1^). Compared with previous reports by Rosa* et al.* [24], who reported 51–143 mg·g^−1^ of total free sugars in the florets of various broccoli cultivars in different seasons, we observed comparatively higher total free sugar in all cultivars. The highest free sugar content was observed in Cheonjae (237.2 mg·g^−1^), followed by Baeridom (205.8 mg·g^−1^) and AMaGi (153.3 mg·g^−1^). In the case of individual free sugars, Cheonjae exhibited statistically higher glucose and fructose content compared with AMaGi and Baeridom (Table 2). However, sucrose, a minor free sugar in broccoli, showed some unusual patterns and exhibited greater difference between florets and leaves. Specfically, AMaGi and Baeridom displayed a greater than 12-fold difference in the sucrose content between florets and leaves, compared with an approximate two fold difference in Cheonjae. 

**Table 2:** Free sugar content (mg·g^−1^ in dry weight) in various parts of broccoli and cauliflower cultivars.

Crop	Cultivar	Part	Glucose	Fructose	Sucrose	Total Free Sugar
Broccoli	AMaGi	Floret	77.4 ^x^ g ^y^	75.3 f	0.6 j	153.3 h
		Leaf	134.5 b	99.3 d	7.8 g	241.6 d
	Baeridom	Floret	105.5 f	98.4 d	1.9 i	205.8 f
		Leaf	112.8 ef	78.2 f	23.3 d	214.3 ef
	Cheonjae	Floret	126.3 b–d	110.2 b	0.7 j	237.2 d
		Leaf	118.9 de	100.8 cd	4.0 h	223.7 e
Cauliflower	Asia purple	Floret	133.3 b	104.1 c	32.2 b	269.6c
		Leaf	127.8 bc	49.4 h	14.8 e	192.0 g
	Asia white	Floret	161.8 a	127.0 a	10.5 f	299.3 b
		Leaf	120.5 c–e	65.1 g	29.2 c	214.8 ef
	Bridal	Floret	133.1 b	113.5 b	72.0 a	318.6 a
		Leaf	127.3 bc	88.3 e	8.8 g	224.4 e

^x^ Values are mean of two independent replications; ^y^ Different letters within the column are statistically significant by Duncan’s multiple-range test at *p* < 0.05.

In cauliflower cultivars, glucose was present in a statistically higher quantity in florets (161.8 mg·g^−1^) than in leaves (120.5 mg·g^−1^) in the Asia white cultivar, whereas Asia purple and Bridal exhibited statistically similar glucose contents in their plant parts. In contrast, all cultivars displayed statistically higher fructose content in florets compared with leaves. Similarly, sucrose content exhibited cultivar- and plant-part-dependent variation. Among the three cultivars and their parts, florets of Asia white showed higher glucose (161.8 mg·g^−1^) and fructose (127.0 mg·g^−1^) content but lower sucrose content (10.5 mg·g^−1^) compared with the other cultivars. Total free sugar content in the florets was statistically higher in Bridal (318.6 mg·g^−1^) than in Asia white (299.3 mg·g^−1^) or Asia purple (269.6 mg·g^−1^), and in all these cultivars, florets exhibited statistically higher total free sugar content compared with leaves (Table 2). The levels observed in this study were lower to those found by Hodges* et al.* [33], who reported approximately 320 mg·g^−1^ of total free sugars in the dry powder of cauliflower florets; however, to the author’s knowledge, this is the first report to examine free sugars in the leaf parts of *Brassica* species. Similar to broccoli, the sucrose content of cauliflower also exhibited greater variation (approximately two- to eightfold) between plant parts. Of the two crops, cauliflower exhibited comparatively higher free sugar content than broccoli. All of these results suggest that the free sugar content in *Brassica* crops is dependent not only on the genotype of the crop, but also on the specific plant tissue.

### 2.4. Fatty Acid Composition

The results of the fatty acid composition analyses are presented in Table 3. Among the 37 fatty acids analyzed, only 13 fatty acids were found in the florets of broccoli cultivars, and only 12 in the leaf parts. Three major fatty acids,* i.e.*, palmitic, linoleic, and linolenic acids, comprised more than 85% of total fatty acids in all cultivars and their parts, except in the florets of AMaGi. The cultivar-dependent variation in major fatty acids was statistically significant only in the florets of some cultivars. The SFA content ranged from 31.51% to 47.81%, with a statistically higher value in florets than in leaves in all cultivars. Monounsaturated fatty acids (MUFA) and polyunsaturated fatty acids (PUFA) ranged from 3.34% to 4.67% and from 47.90% to 65.89%, respectively. Compared with other cultivars, florets of AMaGi exhibited higher SFA (46.75%) and lower PUFA (47.92%). Similarly, in the case of cauliflower cultivars, 12 fatty acids were found, the major ones being palmitic (27.11%–38.42%), linoleic (13.09%–18.97%) and linolenic acid (26.32%–41.03%), accounting for more than 80% of total fatty acid content (Table 3). Similarly enhanced levels of palmitic and linolenic acids were previously reported by Scalzo* et al.* [23]. The other fatty acids were myristic, pentadecanoic, palmitoleic, heptadecanoic, oleic, stearic, arachidic, behenic, and lignoceric acids, and almost all the fatty acids showed statistically significant variation among cultivars and plant parts. Total SFA ranged from 40.08% to 49.70%, with the highest value found in florets of Bridal. MUFA and PUFA ranged from 3.95% to 5.01% and 45.29% to 55.85%, respectively. Compared with leaves, florets exhibited statistically higher SFA values and lower PUFA values in all cultivars. Overall, regardless of crop type and plant part, linolenic acid was the major fatty acid in all cultivars, followed by palmitic and linoleic acids. In both the crop types and cultivars, linolenic acid, which is an important essential fatty acid, was relatively higher in leaves compared to floret parts, thereby suggesting higher nutritional value of leaves because linolenic acid provides various health benefits such as lowering of the plasma lipid level [34]. Altogether, total unsaturated fatty acids were more than 50% of the total fatty acids in all the cases, suggesting the nutritional importance of these crops, as unsaturated fatty acids promote the proper functioning of blood vessels, which in turn reduces the risk of heart attack or stroke [35]. Our results suggest that the fatty acid composition of *Brassica* crops differs depending upon the genotype and plant part, however, further research on *Brassica* fatty acid composition using a large number of germplasms is required. 

**Table 3:** Fatty acid composition (%) in various parts of broccoli and cauliflower cultivars.

Fatty acids	Broccoli						Cauliflower					
	AMaGi		Baeridom		Cheonjae		Asia purple		Asia white		Bridal	
	Floret	Leaf	Floret	Leaf	Floret	Leaf	Floret	Leaf	Floret	Leaf	Floret	Leaf
Lauric (C12:0)	0.17 ^u^ a ^v^	N/D ^w^	0.10 b	N/D	0.10 b	N/D	N/D	N/D	N/D	N/D	N/D	N/D
Myristic (C14:0)	1.04 a	0.39 f	0.51 e	0.28 g	0.39 f	0.29 g	0.87 b	0.91 b	0.87 b	0.68 d	0.77 c	0.67 d
Pentadecanoic (C15:0)	0.40 ab	0.44 a	0.29 e	0.38 bc	0.35 cd	0.36 b–d	0.37 bc	0.30 e	0.36 b–d	0.37 bc	0.32 de	0.44 a
Palmitic (C16:0)	31.74 c	23.55 g	29.75 e	24.05 g	31.03 cd	23.52 g	30.50 de	27.11 f	34.75 b	27.25 f	38.42 a	27.15 f
Palmitoleic (C16:1)	0.66 a	0.21 de	0.44 c	0.21 de	0.37 c	0.13 e	0.73 a	0.22 d	0.53 b	0.17 de	0.40 c	0.20 de
Heptadecanoic (C17:0)	0.61 a	0.40 e	0.30 fg	0.33 f	0.31 fg	0.27 g	0.59 a	0.47 bc	0.51 b	0.45 cd	0.41 de	0.42 de
Stearic (C18:0)	11.90 a	5.53 g	5.98 f	4.70 i	5.59 g	5.07 h	10.37 b	11.72 a	9.97 d	10.11 cd	8.73 e	10.24 bc
Oleic (C18:1n9c)	3.63 d–g	3.13 g	3.82 b–e	3.22 fg	4.30 ab	3.74 c–f	3.56 e–g	3.99 b–e	4.12 a–d	4.23 a–c	4.61 a	3.75 c–f
Linoleic (C18:2n6c)	13.56 ef	14.43 d	18.02 b	14.09 de	18.23 b	14.36 d	16.42 c	14.38 d	16.69 c	14.52 d	18.97 a	13.09 f
Linolenic (C18:3n3)	34.34 f	50.72 a	39.13 de	51.80 a	37.80 e	51.03 a	35.52 f	39.76 cd	30.61 g	41.00 c	26.32 h	42.76 b
Arachidic (C20:0)	0.99 a	0.53 de	0.84 ab	0.42 e	0.74 a–d	0.44 e	0.48 e	0.55 e	0.81 a–c	0.57 c–e	0.61 b–e	0.62 b–e
Behenic (C22:0)	0.30 a	0.21 a–c	0.22 a–c	0.12 c	0.22 a–c	0.28 ab	0.22 a–c	0.23 a–c	0.32 a	0.21 a–c	0.17 b–c	0.23 a–c
Lignoceric (C24:0)	0.66 a	0.46 cd	0.60 ab	0.40 cd	0.57 ab	0.51 bc	0.37 de	0.36 de	0.46 cd	0.44 cd	0.27 e	0.43 cd
SFA ^x^	47.81 b	31.51 g	38.59 f	30.68 g	39.30 e	30.74 g	43.77 c	41.65 d	48.05 b	40.08 e	49.70 a	40.20 e
MUFA ^y^	4.29 bc	3.34 e	4.26 bc	3.43 dc	4.67 ab	3.87 cd	4.29 bc	4.21 bc	4.65 ab	4.40 bc	5.01 a	3.95 c
PUFA ^z^	47.90 f	65.15 a	57.15 b	65.89 a	56.03 c	65.39 a	51.94 e	54.14 d	47.30 f	55.52 c	45.29 g	55.85 c

^u^ Values are the mean of two independent replications; ^v^ Different letters within the raw are statistically significant by Duncan’s multiple-range test at *p* < 0.05; ^w^ N/D: Not detected; ^x^ SFA: saturated fatty acid; ^y^ MUFA: monounsaturated fatty acid; ^z^ PUFA: polyunsaturated fatty acid.

### 2.5. Antioxidant Activity

In our experiment, we measured antioxidant activity by evaluating the 2,2,-diphenyl-1-picrylhydracyl (DPPH) radical-scavenging activity of different concentrations of methanolic extracts from *Brassica* crops. The IC_50_ (50% of inhibition) value was calculated following a linear regression analysis of the observed inhibition percentage* versus* concentration, where a lower IC_50_ value shows higher antioxidant activity. Antioxidant activity in broccoli cultivars varied significantly in florets, whereas no such variation was observed in leaves (Figure 1D). Both AMaGi and Cheonaje showed relatively higher antioxidant activity in florets compared with leaves, whereas Baeridom showed the opposite result. Of all cultivars and plant parts, the highest antioxidant activity was observed in floret of Cheonjae cultivar (IC_50_ value = 2.27 mg·mL^−1^). In the case of cauliflower, only the Asia purple showed statistically higher antioxidant activity in its floret (IC_50_ value = 1.12 mg·mL^−1^) compared with leaves (IC_50_ value = 2.27 mg·mL^−1^), while other cultivars exhibited higher antioxidant activity in their leaf parts than in floret parts. This might be due to the higher content of total phenols, as phenolics are major contributors to total antioxidant activity [36].

Overall, antioxidant activity differed depending upon the crop type, cultivar, and plant part in most cases. However, of all cultivars and plant parts, florets of Asia purple showed the lowest IC_50_, thus indicating the highest antioxidant activity (Figure 1D) and enhanced potential health benefits of these cultivars.

### 2.6. Correlations among Phytonutrients

To determine the contribution of antioxidants to antioxidant activity and phytonutrients, we performed a correlation analysis on the relationships among vitamin C, phenolics, flavonoids, total glucosinolates, and antioxidant activity (Table 4). In our study, regardless of the crop type, cultivar or plant part, vitamin C exhibited the highest positive correlation with total flavonoid (*r* = 0.574 **), followed by the correlation with total phenol (*r* = 0.522 **) and total glucosinolates (*r* = 0.494 **). Likewise, we found a significant positive correlation between antioxidant activity and total phenols (*r* = 0.698 **), vitamin C (*r* = 0.632 **), and total flavonoid (*r* = 0.456 **). These results are consistent with previous reports that described the antioxidant capacity of various *Brassica* vegetables [6,20,30,37]. In contrast, no correlation was found between antioxidant activity and total glucosinolates, perhaps due to the low antioxidant capacity of glucosinolates or the low quantity of glucosinolates in these cultivars. The stronger positive correlation between antioxidant activity and total phenol content found in this study is in agreement with Aires* et al.* [6] and Samec* et al.* [37], possibly due to the greater contribution of phenolic compounds to antioxidant activity.

**Table 4:** Correlation coefficients among phytochemicals and antioxidant activities.

Attributes	Total Phenol	Total Flavonoid	Total Glucosinolate	Antioxidant Activity
Vitamin C	0.522 **	0.574 **	0.494 **	0.632 **
Total phenol	1	0.292	0.169	0.698 **
Total flavonoid	0.292	1	0.014	0.456 **
Total glucosinolate	0.169	0.014	1	0.219

** Correlation is significant at *p* < 0.01.

## 3. Experimental Section

### 3.1. Plant Materials

Six commercial F_1_ hybrid *Brassica* cultivars, including three cauliflower cultivars (Asia purple, Asia white, and Bridal) and three broccoli cultivars (AMaGi, Baeridom, and Cheonjae), were used in this study. All were grown in an experimental field of the National Institute of Horticultural and Herbal Science (NIHHS), Rural Development Administration (RDA), Suwon, South Korea. For both the crop types, seedlings were transplanted to the cultivation field 33 days after sowing. Seedlings were planted in rows with 50 cm between plants and 60 cm in rows. The sowing date was 5 March 2011, and the plant materials were harvested at commercial maturity stage, which occurred between 65 and 75 days after sowing. During the field experiments, water, fertilizers and pesticides were applied according to standard cultural practices of NIHHS and RDA. Ten broccoli/cauliflower heads were used for each sample. After harvesting, florets and leaf parts were separated, cut into small pieces, and then freeze dried. The samples were ground into fine powder and then stored at −20 °C until used for the analysis of glucosinolates, vitamin C, total phenol, total flavonoid and free sugar content, fatty acid composition, and antioxidant activity.

### 3.2. Authentic Standards and Chemicals

The glucosinolate standards, glucoiberin, progoitrin, glucoraphanin, sinigrin, gluconapin, glucobrassicanapin, glucoerucin, glucobrassicin, and gluconasturtiin, were purchased from Cfm Oskar Co. (Marktredwitz, Germany). Authentic standards for DEAE (Diethyl aminoethyl) Sephadex-A25, aryl sulfatase (EC 3.1.6.1, type H-1) from *Helix pomatia*, vitamin C, glucose, sucrose, fructose, gallic acid, DPPH (2,2-diphenyl-1-picrylhydrazyl), and catechin hydrate were purchased from Sigma-Aldrich (St. Louis, MO, USA). A standard for fatty acid methyl esters (FAME) was obtained from Supelco (Bellefonte, PA, USA). Chemicals such as sodium hydroxide, sodium carbonate, sodium nitrite, aluminum chloride, Folin−Ciocalteu reagent, and 2,2-dimethoxypropane were purchased from Sigma-Aldrich (St. Louis, MO, USA). Benzene, n-heptanes, and sulfuric acid were acquired from Daejung Chemicals (Seoul, Korea). Other chemicals, including acetonitrile (HPLC grade), methanol (HPLC grade), and formic acid (ACS reagent), were purchased from J. T. Baker (Phillipsburg, NJ, USA).

### 3.3. Analysis of Vitamin C

Vitamin C content was analyzed according to the methods described by Spinola* et al.* [38] with modifications. Freeze-dried powdered *Brassica* samples (0.5 g) were extracted with 5% metaphosphoric acid solution. Then, after centrifugation and filtration (with a 0.20 μm syringe filter), the aliquot was analyzed using an H-Class UPLC (ultra performance liquid chromatography) (Waters, Milford, MA, USA) equipped with an Acquity UPLC^®^ HSS T3 (2.1 × 100 mm, 1.8 μm, Waters) column and PDA (photo diode array) detector (Waters) at 254 nm in wave length. An isocratic mobile phase composed of aqueous 0.1% (*v*/*v*) formic acid at a flow rate of 0.3 mL·min^−1^ was used for separation of the vitamin C peak. An authentic ascorbic acid standard at various concentrations (5, 10, 25, 50, and 100 ppm) was used for the identification and quantification of the peak and vitamin C content was expressed as milligrams per 100 gram (mg·100 g^−^^1^) of dry weight.

### 3.4. Analysis of Total Phenol

Total phenolic content was estimated by the Folin–Ciocalteu colorimetric method based on the procedure previously described by Singleton and Rossi [39] using gallic acid as a standard phenolic compound. Briefly, 1 g freeze-dried powdered sample was extracted in 80% methanol for 15 h at room temperature on an orbital shaker. Then, the extract was centrifuged and filtered through a Whatman No. 42 filter paper, and 1 mL of supernatant was mixed with 3 mL distilled water in a 15 mL falcon tube. After adding 1 mL of Folin reagent, the solution was incubated in a water bath at 27 °C for 5 min. Then, 1 mL of saturated sodium carbonate was added. After 1 h, absorbance of the extract was measured with an EON^TM^ microplate spectrophotometer (BioTek^®^ Instruments Inc. Highland Park, Winooski, VT, USA) at 640 nm. Gallic acid standards at different concentrations (5, 10, 25, 50, 75, and 100 ppm) were used for the calibration. Total phenol content was expressed as milligrams of gallic acid equivalent per 100 g (mg·GAE 100 g^−^^1^) dry weight. 

### 3.5. Analysis of Total Flavonoid

The vegetable extracts obtained for total phenol analysis were also used for total flavonoid analysis using a colorimetric method described by Zhishen* et al.* [40]. Only 1 mL of the extract was kept in a 15 mL Falcon tube containing 4 mL of distilled water, and then 0.3 mL of 5% sodium nitrite was added. After 5 min, 10% of AlCl_3_ was added to the solution. At 6 min, 2 mL of 1 M NaOH was added, and the sample was brought to final volume 10 mL using distilled water. The solution was mixed carefully, and the absorbance was measured at 510 nm using an EON^TM^ microplate spectrophotometer (BioTek^®^ Instruments Inc., Highland Park, Winooski, VT, USA). Catechin hydrates at different concentrations (5, 10, 25, 50, 75, and 100 ppm) were used as the standard compound. Total flavonoid was expressed as milligrams of catechin hydrate equivalent per 100 g (mg·CE 100 g^−^^1^) of dry weight.

### 3.6. Analysis of Glucosinolates

Sample preparation and glucosinolate analysis were performed according to methods described by Lee* et al.* [41]. Briefly, freeze-dried powder samples (0.1 g) were extracted with 2 mL of boiling methanol (70%) for 20 min and centrifuged at 12,000 rpm for 10 min at 4 °C, after which the pellet was re-extracted one more time and the supernatants were combined. The crude glucosinolate extract was then loaded onto a Mini Biospin chromatography column (Bio-Rad Laboratories, Hercules, CA, USA) containing 0.5 mL of DEAE-Sephadex A 25, which was preactivated with 0.1 M sodium acetate (pH 4.0), following which desulfation was carried out by the addition of 200 µL of purified aryl sulphatase*.* The column was capped and left for 24 h at room temperature, and the desulfoglucosinolates were eluted with 1.5 mL distilled water, filtered through a 0.2-µm syringe filter, injected into H-Class UPLC (Waters) using an Acquity UPLC^®^ BEH-C18 column (1.7 µm, 2.1 × 100 mm; Waters), and measured at 229 nm with a PDA detector. Solvent A (100% distilled water) and solvent B (20% acetonitrile) were used for the elution of compounds at the flow rate of 0.2 mL·min^−1^. The gradient programs were as follows: a linear step from 1% to 99% of solvent B within 6 min, followed by constant conditions for up to 10 min and then a quick dropdown to 1% of solvent B at 12 min and isocratic conditions with 1% of solvent B up to 15 min. Authentic standards of glucosinolates were used for the identification and quantification of the peaks. Total and individual glucosinolates were expressed as micromole per gram (µmol·g^−1^) of dry weight.

### 3.7. Analysis of Free Sugar

Free sugars (glucose, fructose, and sucrose) were analyzed according to Bhandari* et al.* [42] with some modifications. Briefly, powdered *Brassica* samples (1.0 g) were extracted with distilled water by shaking for 20 min in a water bath at 80 °C, centrifuged at 3500 rpm for 5 min, filtered through a 0.45 µm syringe filter, and analysed using an HPLC system (Waters) with a Kromasil^®^ 100-NH2 column (250 × 4.6 mm; Eka Chemicals AB, Seperation Products, Bohus, Sweden) and RI (refractive index) detector (Waters) with acetonitrile/distilled water (75/25, *v*/*v*) for the mobile phase at a flow rate of 1.5 mL·min^−1^. Authentic standards of free sugars at different concentrations were used for the identification and quantification of the peaks. Total as well individual free sugars were expressed as milligram per gram (mg·g^−1^) of dry weight.

### 3.8. Analysis of Fatty Acid Composition

Samples for fatty acid composition analyses were prepared and analyzed according to Bhandari* et al.* [42]. Briefly, powdered samples (0.1 g) were mixed with 680 µL of methylation mixture (MeOH: benzene: 2,2-dimethoxypropane: H_2_SO_4_ = 39:20:5:2) and 400 µL of heptane. After vigorous mixing and heating for 2 h at 80 °C in a water bath, samples were cooled at room temperature, and then a heptane layer was collected and injected into a gas chromatograph (GC; Varian 3800, Palo Alto, CA, USA) equipped with a flame ionization detector and a capillary column: CP SIL 88 CB FAME (50 m × 0.25 mm, 0.25 µm, Agilent Technologies, Santa Clara, CA, USA). The temperature was set 210 °C for both the injector and FID (flame ionization detection) detector (Varian 3800, Palo Alto, CA, USA). The injection volume was 1 µL with a split ratio of 1:50 on a constant column flow (1 mL·min^−1^) of helium gas. The oven temperature was initially maintained at 100 °C for 5 min, and FID was increased up to 160 °C at a rate of 5 °C min^−1^, maintained for 5 min, and then increased again at a rate of 4 °C min^−1^ up to 180 °C. All the fatty acids were expressed as relative percentage (%) of dry weight.

### 3.9. Determination of Antioxidant Activity

The antioxidant activity of vegetable extracts taken from different plant parts was determined using the 2,2,-diphenyl-1-picrylhydracyl (DPPH) radical scavenging method described in Koleva* et al.* [43], with modifications. The aliquot obtained for total phenol analysis was also used for the measurement of radical scavenging activity. At first, 400 μM of DPPH solution in 80% MeOH was prepared. Then, 100 μL of DPPH solution was added to 100 μL of different concentrations of extracts (0.5, 1.0, 1.5, 2.5, and 5.0 mg·mL^−^^1^) and 100 μL of methanol in 96-well plates. After 30 min, the absorbance of the resultant solution was measured using an EON^TM^ microplate spectrophotometer (BioTek^®^ Instruments Inc., Highland Park, Winooski, VT, USA) at 517 nm wavelength against a blank, which was 80% methanol without DPPH. Similarly, the absorbance of samples was measured after mixing 100 μL samples with 100 μL of 80% methanol. Free radical-scavenging activity (%) was calculated using the following equation:

% of DPPH radical-scavenging activity = (B − A) × 100/B

where A is the absorbance of [(Sample + DPPH) − (Sample + Methanol)], and B is the absorbance of [(Methanol + DPPH) − (Methanol)].

The IC_50_ value, which is the concentration required to obtain 50% antioxidant capacity, was calculated and used to compare the antioxidant activity of sample extracts.

### 3.10. Statistical Analyses

For each sample, three independent replicate measurements were used in all statistical analyses. To determine differences among crop types, cultivars, and plant parts, one-way analysis of variance (ANOVA) followed by Duncan’s multiple-range test (DMRT) was performed at a significance level of 0.05 using SAS^®^ 9.2 software (SAS Institute Inc., Cary, NC, USA, 2013).

## 4. Conclusions

In conclusion, the results of our study showed that the levels of chemical constituents and antioxidant activity are significantly dependent on crop type, cultivar, and plant part. Most of the compounds as well as the antioxidant activity were higher in plant florets; however, phytonutrients, such as flavonoids in both broccoli and cauliflower and free sugar in broccoli cultivars, revealed statistically higher values in leaves, indicating that leaves are a good source of those phytochemicals. Similarly, the highest levels of vitamin C, total phenol, total flavonoids, free sugar, and antioxidant activity were observed in the cauliflower cultivars, whereas the highest total glucosinolate was present in the broccoli cultivars; however, no specific cultivar had significantly higher quantities of all the phytochemicals. All of these results suggest that phytonutrients in *Brassica* are affected in different ways depending on the nature of the compounds. Furthermore, the major fatty acids were palmitic, linoleic, and linolenic acids with a high compositional ratio of unsaturated fatty acids, thereby signifying the nutritional value of these fatty acids that are responsible for the promotion of human health in a number of different ways. 
